# Editorial: Intranasal Delivery of Central Nervous System Active Drugs: Opportunities and Challenges

**DOI:** 10.3389/fphar.2022.927812

**Published:** 2022-06-14

**Authors:** Márcio Rodrigues, Javed Ali, Gilberto Alves

**Affiliations:** ^1^ CICS-UBI—Health Sciences Research Centre, University of Beira Interior, Covilhã, Portugal; ^2^ CPIRN-UDI-IPG—Center for Potential and Innovation of Natural Resources, Research Unit for Inland Development, Polytechnic Institute of Guarda, Guarda, Portugal; ^3^ Department of Pharmaceutics, School of Pharmaceutical Education and Research, New Delhi, India

**Keywords:** brain targeting, central nervous system, intranasal drug delivery, nasal route, pharmacokinetics, pharmacodynamics

Over the last years there has been a growing interest in intranasal delivery of drugs for disorders affecting the central nervous system (CNS). In fact, the drug treatment of CNS disorders is often a challenging task due to multiple factors that have a negative impact on an effective brain targeting, with poor blood-brain barrier (BBB) permeability being perhaps the most relevant one. Nowadays, it is well-known that drugs can reach the brain noninvasively, bypassing the BBB, after intranasal administration. Thus, the intranasal route represents an attractive opportunity to improve the treatment of multiple sclerosis, Alzheimer’s disease, Parkinson’s disease, epilepsy, psychosis, central pain, brain cancer, and many other CNS disorders (Erdő et al., 2018; Islam et al., 2020).

Nasal drug delivery offers many advantages over conventional systemic delivery routes, such as its non-invasive character, ease of administration, a fast onset of action, and in many cases reduced side effects due to a more targeted delivery at a low dose, enabling to achieve a high benefit-risk relationship (Pires et al., 2022). Thus, intranasal delivery affords patient comfort and compliance that are hurdled by parenteral drug therapy and also could result in faster systemic drug absorption than the oral route (Fortuna et al., 2014).

Bearing in mind all the aforementioned aspects, the direct nose-to-brain delivery has emerged as a promising strategy to circumvent the BBB and to deliver drugs to the brain (Pires et al., 2022). Indeed, this strategy has been fruitful as exemplified by some nasal formulations containing benzodiazepines that have recently reached the market to be used in conditions that require an acute treatment (Nayzilam^®^ and Valtoco^®^). More specifically, Nayzilam^®^ is a midazolam nasal spray (5 mg per dose) that was developed by Proximagen, Ltd., Cambridge, and is indicated for the acute treatment of seizure clusters. On the other hand, Valtoco^®^ is a diazepam formulation (5, 10, 15 or 20 mg per dose), developed by Neurelis, Inc., CA, which is indicated for the acute treatment of intermittent, stereotypic episodes of frequent seizure activity (i.e., seizure clusters and acute repetitive seizures) that are distinct from a patient’s usual seizure pattern in patients with epilepsy 6 years of age and older (Cartt et al., 2012; U.S. Food and Drug Administration, 2019; U.S. Food and Drug Administration, 2020). These formulations are efficacious, safe and also well-tolerated, but some adverse reactions were also observed such as nasal discomfort, throat irritation, rhinorrhea and alteration in taste (Agarwal et al., 2013; Maglalang et al., 2018; U.S. Food and Drug Administration, 2019; Hogan et al., 2020). Accordingly, it is expected that research in this field still has a long way to go, and we are sure that the number of CNS active drugs that can be administered intranasally will increase in the future.

From the published studies in this Research Topic, one review is highlighted by Wu et al. with a scientometric analysis regarding intranasal delivery research in which a comprehensive knowledge map, a development landscape and the future directions of intranasal delivery research were addressed, providing a practical and valuable reference for scholars and policymakers with interest in this area. In an another review conducted by Pandey et al. the novel strategies that could lead to an improved efficacy of CNS agents using several advanced drug delivery systems by intranasal route have been discussed. Furthermore, preclinical and clinical advancements on the delivery of antipsychotics using intranasal route have also been emphasized.

In one of the experimental studies, the study conducted by Micheli et al. demonstrated in rats the potential of the intranasal administration of a low-dose naltrexone (an opioid antagonist) to counteract morphine and oxycodone induced gastrointestinal and CNS side effects, without impairing opioid analgesia. However, these promising results require further in-depth studies for a possible clinical use.

Moreover, in an another experimental study conducted by Borroto-Escuela et al., it was demonstrated the intranasal delivery of galanin 2 and neuropeptide Y1 agonists directly to CNS. These two agonists interact with neuropeptide Y1 receptor and galanin receptor 2 enhancing spatial memory performance and neuronal precursor cells of the dentate gyrus proliferation in the dorsal hippocampus in rats. This study shows a promising novel route of administration for treatment of neurodegenerative disorders as the Alzheimer’s disease.

Solés-Tarrés et al. demonstrated that the intranasal administration of pituitary adenylate cyclase-activating polypeptide (PACAP) to the R6/1 mouse model of Huntington’s disease restored the motor function and increased the striatal levels of PACAP-selective PAC1 receptor (PAC1R), cyclic-adenosine monophosphate response element-binding protein, and brain-derived neurotrophic factor. This effect in Huntington’s disease striatum allows the recovery of motor function and point out PAC1R as a therapeutic target for treatment of Huntington’s disease.

In a global perspective, these literature reviews and experimental studies reinforce the potential of intranasal route to treat several CNS conditions and will go a long way to demonstrate the clinical usage of intranasal delivery.
